# Nécrose du membre inférieur chez un adolescent : à propos d’un cas négligé d’envenimation ophidienne dans un hôpital rural du sud Togo

**DOI:** 10.48327/mtsi.v6i3.2026.559

**Published:** 2026-07-13

**Authors:** Kossi Albert LABOU, Tamassi Bertrand ESSOBIYOU, Anani Komla AWUDZA, Abla Gildas TENOU

**Affiliations:** 1 Service de chirurgie générale, CHU Sylvanus Olympio, Lomé, Togo; 2 Service de chirurgie générale, Hôpital Saint Jean de Dieu Hospital, Afagnan, Togo; 3 Service de pédiatrie, Hôpital Saint Jean de Dieu Hospital, Afagnan, Togo

**Keywords:** Envenimation ophidienne, Enfants, Tradithérapie, Gangrène, Amputation, Togo, Afrique subsaharienne, Snakebite, Children, Traditional medicine, Gangrene, Amputation, Togo, Sub-Saharan Africa

## Abstract

**Introduction:**

L’envenimation est un problème majeur de santé publique sous-estimé en Afrique subsaharienne. Le retard de consultation et le manque d’antivenin sont les principaux problèmes qui limitent une prise en charge correcte. Nous rapportons le cas d’un patient traité dans un hôpital rural du sud Togo pour une gangrène humide de la jambe droite compliquant une envenimation négligée.

**Observation:**

Un adolescent togolais de 12 ans, sans antécédents pathologiques connus, a été admis aux urgences pour une plaie chronique de la jambe droite. L’anamnèse a révélé qu’il avait été mordu par un serpent un mois plus tôt alors qu’il travaillait dans les champs, et qu’il avait été traité par la médecine traditionnelle. L’examen a révélé une gangrène humide étendue de la jambe droite, avec exposition des deux os de la jambe. Une amputation transfémorale a été réalisée. L’évolution post-opératoire a été favorable.

**Conclusion:**

La prise en charge des envenimations est difficile dans notre pays en raison des consultations tardives et faute d’un traitement approprié.

## Abstract (en)

**Introduction:**

Snakebite is a significant yet underestimated public health issue in sub-Saharan Africa. Delayed presentation and a lack of antivenom are the main factors that limit appropriate management. Here, we present the case of a patient who was treated at a rural hospital in southern Togo for wet gangrene of the right leg resulting from a neglected snakebite.

**Observation:**

A 12-year-old Togolese adolescent with no known medical history was admitted to the emergency department for treatment of a chronic wound on his right leg. The questioning revealed that he had been bitten by a snake one month earlier while working in the fields and had been treated with traditional medicine. Physical examination revealed extensive wet gangrene of the right leg, with both leg bones exposed. A transfemoral amputation was performed. The postoperative course was favorable.

**Conclusion:**

Managing snakebites is challenging in our country due to delayed presentation and a lack of appropriate treatment.

## Introduction

L’envenimation ophidienne est un problème majeur de santé publique en régions tropicales et subtropicales où elle constitue une véritable urgence médico-chirurgicale [2,3,9]. La morbimortalité y est élevée en raison de sa fréquence et de l’insuffisance de sa prise en charge. Nous rapportons le cas d’une gangrène humide de jambe avec fonte tissulaire secondaire à une envenimation ophidienne négligée chez un adolescent, ayant nécessité une amputation au niveau de la cuisse.

## Observation

Un adolescent de 12 ans a été reçu en consultation chirurgicale à l’hôpital Saint Jean de Dieu d’Afagnan (Région maritime, sud Togo) le 3 juillet 2024 pour une gangrène de la jambe droite. La lésion était consécutive à une morsure de serpent au pied droit survenue un mois auparavant à Dzrekpo (environ 7 km au sudouest d’Afagnan). Mordu audessus de la malléole externe droite au cours de travaux champêtres, le patient n’a informé ses parents que devant l’aggravation de la douleur et la survenue d’un oedème du membre. Il a signalé avoir ressenti une sorte de piqûre sur le chemin de retour sans précision sur la nature de la morsure. La morsure de serpent a été suspectée devant deux points d’entrée identifiés ultérieurement.

L’enfant a été pris en charge initialement par un tradipraticien qui a pratiqué des scarifications et appliqué des plantes médicinales de nature ignorée. L’évolution s’est faite vers la régression de l’oedème et la survenue d’une plaque de nécrose autour de la zone de morsure avec extension progressive et ascendante. Devant l’aggravation des lésions, les parents ont décidé d’amener le patient dans notre centre pour une meilleure prise en charge. Aucune pose de garrot n’a été réalisée ni par la famille ni par le tradipraticien. Le patient présentait un bon état général avec une stabilité hémodynamique. L’examen du membre retrouvait une fonte purulente des parties molles de la jambe droite avec exposition des deux os de ladite jambe étendue aux 2/3 distaux (Fig. 1). Le bilan biologique réalisé en urgence comprenait la numération formule sanguine (NFS), le bilan rénal (urémie et créatinémie) et le bilan de coagulation (test de coagulation sur tube sec, taux de prothrombine et temps de céphaline activé). La NFS retrouvait une anémie avec un taux d’hémoglobine à 7,8 g/dl, une leucocytose à 17,8 G/l avec une neutrophilie à 13 G/l, un taux de plaquettes à 177 G/l. Le bilan rénal était normal. Il n’y avait pas de troubles de la coagulation.

**Figure 1:**
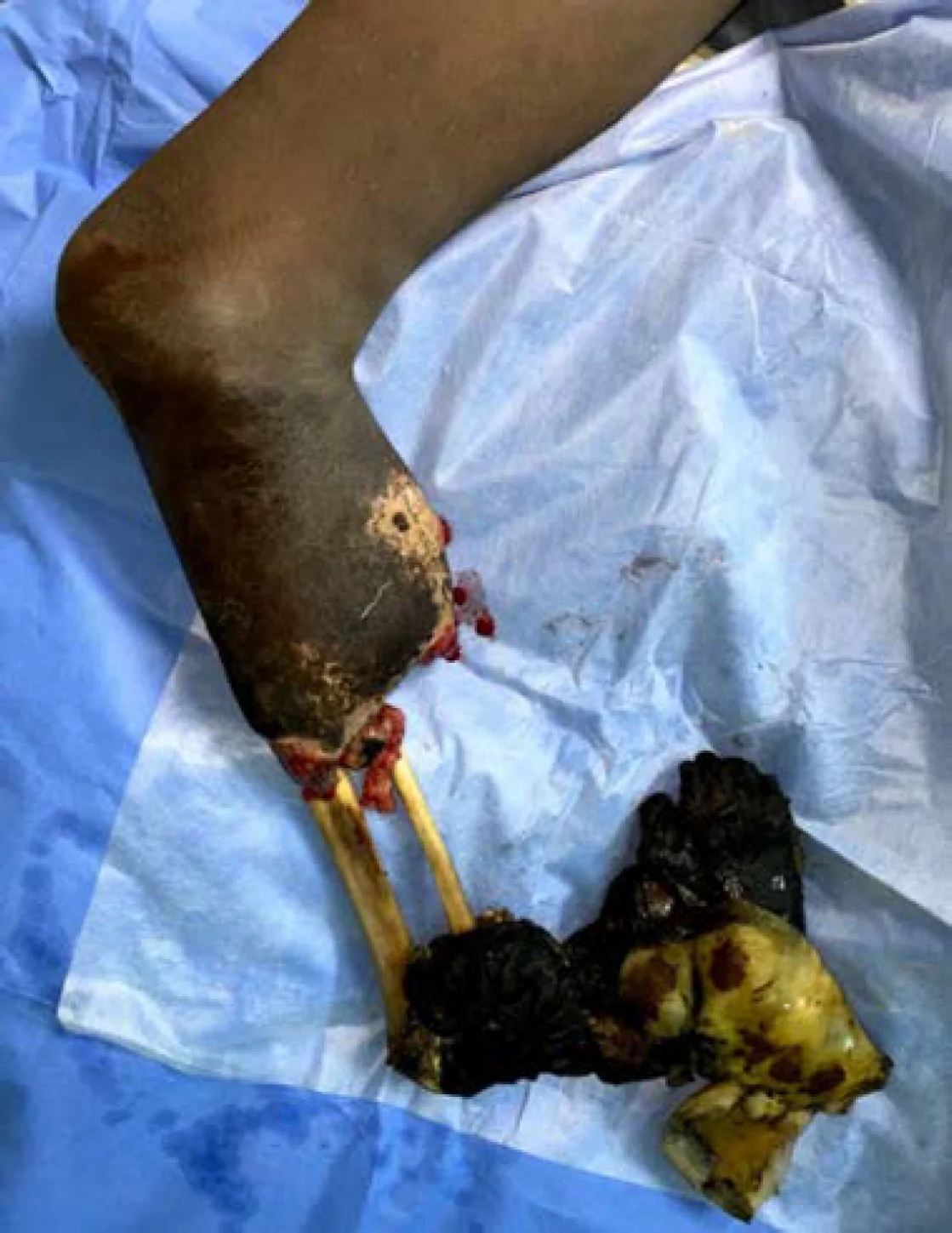
Gangrène de jambe avec fonte tissulaire et exposition des deux os de la jambe

Nous avons conclu à une gangrène humide de la jambe droite compliquant une envenimation négligée et avons indiqué une amputation transfémorale du membre pelvien droit. Une antibiothérapie pré-opératoire associant de la ceftriaxone et du métronidazole a été initiée. Après une transfusion pré-opératoire de deux poches de culot globulaires (250 ml chacune) et d’une poche de plasma frais congelé (125 ml), le patient a été amputé. Les suites opératoires immédiates ont été simples avec reverticalisation du patient par des cannes anglaises en attente d’une prothèse de jambe. Le contrôle post-opératoire à un mois montrait une bonne cicatrisation de la plaie opératoire. Le patient a été adressé au centre national pour l’appareillage.

## Discussion

Les envenimations ophidiennes sont classées dans les maladies tropicales négligées [10]. En Afrique subsaharienne, elles sont essentiellement dues à deux familles de serpents : les Viperidae (*Bitis* spp., *Echis* spp., *Atheris* spp., *Causus* spp.) et les Elapidae (*Naja* spp. et *Dendroaspis* spp.) [1,2,3,6]. Les premiers sont responsables d’envenimations inflammatoires, hémorragiques et nécrosantes [2,11]. Les seconds entraînent des troubles neurologiques (pouvant aller jusqu’à la paralysie respiratoire) et, plus rarement, des nécroses [4,11]. Au Togo, plusieurs études ont montré que les morsures étaient occasionnées majoritairement par les Viperidae et beaucoup plus rarement par les Elapidae [2,3,7,11].

L’intérêt de cette étude est de rappeler la gravité des envenimations ophidiennes. L’évolution vers des séquelles lourdes et un handicap majeur dans ce cas, s’explique par le retard de consultation et un parcours thérapeutique probablement complexe mais que cet adolescent n’a pas détaillé.

L’évolution nécrotique est compatible avec une envenimation par un Viperidae. Il pourrait s’agir d’*Echis ocellatus*, espèce fréquente au Togo, de petite taille et s’enfuyant rapidement, ce qui pourrait expliquer que le serpent n’ait pas été vu. L’éventualité d’une morsure par *Bitis arietans* ou *Naja nigricollis*, tous deux également responsables de nécrose, est peu probable en raison de la taille de ces serpents et de leur relative lenteur à s’enfuir, notamment *B. arietans*.

L’enfant n’a pas eu de garrot, ce qui exclut l’hypothèse d’une gangrène due à la pose d’un garrot serré et maintenu en place trop longtemps. L’anamnèse difficile à reconstituer n’évoque pas de saignements pourtant habituels à la suite d’une morsure par *E. ocellatus*. Le retard considérable de consultation explique l’évolution vers une nécrose de la jambe ayant nécessité l’amputation. L’antivenin, seul traitement étiologique des envenimations, apporte des anticorps de défense qui neutralisent et éliminent le venin de l’organisme. Cette action contribue à réduire la durée d’exposition aux effets cytotoxiques et à éviter l’installation de la nécrose [5]. Cependant, le venin disparaît spontanément de l’organisme en moins d’une semaine après la morsure. À l’arrivée du patient à l’hôpital, un mois après la morsure, l’administration d’antivenin n’aurait apporté aucun bénéfice et était donc inutile.

Au Togo, ainsi que dans la plupart des pays d’Afrique subsaharienne, le système de santé est confronté à des ruptures fréquentes de stock d’antivenin en raison de leur coût élevé qui les rend indisponibles dans les structures périphériques [7,8,11]. À cela s’ajoute la prédominance d’une prise en charge traditionnelle initiale privilégiée par la population en raison de croyances ancestrales, de la difficulté d’accès aux centres de santé dans ces zones rurales et de son faible coût [2,7,11]. Ces traitements traditionnels, utilisant scarifications et décoctions, favorisent la surinfection des lésions et compromettent ainsi le pronostic des envenimations [4].

## Conclusion

L’envenimation ophidienne nécessite une prise en charge rapide et adéquate. Les populations ont recours le plus souvent au traitement traditionnel; cette pratique entraîne un retard de consultation qui se traduit par la survenue des graves complications. L’immunothérapie antivenimeuse reste la seule alternative à condition d’être administrée le plus tôt possible après la morsure. Il est impératif de mener des politiques visant à la rendre accessible dans toutes les structures et à en réduire le coût.

## Considérations éthiques

Un consentement éclairé et signé a été obtenu de la part du père du patient pour la publication de ses données médicales.

## Financement

Aucun financement n’a été obtenu pour la réalisation de ce travail.

## Contribution des auteurs

Conception de l’étude, collectes des données et images : LKA, ETB

Rédaction du manuscrit : LKA, TGA

Révision du manuscrit : ETB, AKA, TGA

Tous les auteurs ont lu et approuvé la version finale du manuscrit.

## Déclaration de liens d’intérêt

Aucun lien d’intérêt n’a été déclaré.
