# Supplementary material for: Lower limb necrosis in an adolescent: a report on an overlooked case of snakebite at a rural hospital in southern Togo
Source: Med Trop Sante Int. 2026 Jul 13;6(3):mtsi.v6i3.2026.559. [Article in French] doi: 10.48327/mtsi.v6i3.2026.559 (PMC13697903; doi:10.48327/mtsi.v6i3.2026.559)
Supplement: Related texts

# Research article: Lower limb necrosis in an adolescent: a report on an overlooked case of snakebite at a rural hospital in southern Togo

## Introduction

Snakebite is a significant public health concern in tropical and subtropical regions, where it is considered a medical and surgical emergency [2,3,9]. These regions have high morbidity and mortality rates due to the frequency of the condition and inadequate management. Here, we present a case of wet gangrene of the leg with tissue necrosis secondary to neglected snakebite in an adolescent that required thigh amputation.

## Case report

On July 3, 2024, a 12-year-old adolescent was admitted to the surgical outpatient clinic at Saint Jean de Dieu Hospital in Afagnan (Maritime Region, southern Togo) for gangrene of the right leg. The lesion resulted from a snakebite to the right foot that occurred one month earlier in Dzrekpo, approximately seven kilometers southwest of Afagnan. The patient was bitten above the right lateral malleolus while working in the fields but did not inform his parents until the pain worsened and swelling developed in the limb. He reported feeling a sort of sting on his way home, but he did not specify the nature of the bite. A snakebite was suspected based on two entry points that were later identified.

Initially, the child was treated by a traditional healer who performed scarification and applied medicinal plants of unknown origin. The swelling resolved and a necrotic patch developed around the bite site with progressive and ascending spread. As the lesions worsened, the parents decided to bring the patient to our center for more appropriate care. Neither the family nor the traditional healer applied a tourniquet.

The patient was in good general condition and hemodynamically stable. Examination of the right leg revealed purulent soft-tissue breakdown with exposure of the two bones extending to the distal two-thirds (Fig. 1). The emergency laboratory workup included a complete blood count (CBC), renal function tests (urea and creatinine levels), and coagulation tests (whole blood clotting test, prothrombin time, and activated partial thromboplastin time). The CBC showed anemia, with a hemoglobin level of 7.8 g/dL; leukocytosis, with a white blood cell count of 17.8 G/L and a neutrophil count of 13 G/L; and a platelet count of 177 G/L. Renal function tests were normal. There were no coagulation disorders.

**Figure 1:** Gangrene of the leg with tissue necrosis and exposure of both leg bones

We diagnosed wet gangrene of the right leg, which resulted from a neglected envenomation, and recommended a transfemoral amputation of the right lower extremity. We initiated preoperative antibiotic therapy combining ceftriaxone and metronidazole. After receiving a transfusion of two units of packed red blood cells (250 mL each) and one unit of fresh frozen plasma (125 mL), the patient underwent amputation. The postoperative course was uneventful, and the patient regained mobility with the use of crutches while awaiting a prosthetic leg. The one-month postoperative follow-up showed good healing of the surgical wound. The patient was referred to the national prosthetics center.

## Discussion

Snakebite is classified as a neglected tropical disease [10]. In sub-Saharan Africa, they are primarily caused by two families of snakes: the Viperidae (*Bitis* spp., *Echis* spp., *Atheris* spp., *Causus* spp.) and the Elapidae (*Naja* spp. and *Dendroaspis* spp.) [1,2,3,6]. The former cause inflammatory, hemorrhagic, and necrotizing envenomations [2,11]. The latter lead to neurological disorders, which can progress to respiratory paralysis, and necrosis, though this is rare [4,11]. In Togo, several studies have shown that bites are primarily caused by Viperidae and much less frequently by Elapidae [2,3,7,11].

The purpose of this study is to emphasize the severity of snakebites. In this case, the progression to severe sequelae and major disability can be explained by the delay in seeking medical care and the likely complex course of treatment, which the adolescent did not detail.

The necrotic progression of the disease is consistent with envenomation by a Viperidae species. One possibility is *Echis ocellatus*, a small, fast-fleeing species common in Togo that might explain why the snake was not seen. A bite from *Bitis arietans* or *Naja nigricollis* is unlikely due to the size of these snakes and their relatively slow speed when fleeing, particularly *B. arietans.* The child did not have a tourniquet applied, ruling out gangrene caused by a tight tourniquet left in place too long. The difficult-to-reconstruct medical history does not mention any bleeding, though this is typical following a bite from *E. ocellatus*. The considerable delay in seeking medical attention explains the progression to necrosis of the leg, which necessitated amputation. Antivenom is the only etiological treatment for snakebites; it provides defensive antibodies that neutralize and eliminate the venom from the body. This helps reduce exposure to cytotoxic effects and prevents necrosis [5]. However, the venom clears from the body within a week of the bite. Since the patient arrived at the hospital a month after being bitten, administering antivenom was unnecessary.

In Togo, as in most sub-Saharan African countries, the healthcare system frequently experiences shortages of antivenom due to its high cost. This makes it unavailable in peripheral healthcare facilities [7,8,11]. Additionally, traditional initial treatment is prevalent due to ancestral beliefs, the difficulty of accessing health centers in rural areas, and the low cost of these treatments [2,7,11]. These traditional treatments involve scarification and herbal decoctions and promote secondary infection of the wounds, thus compromising the prognosis for snakebites [4].

## Conclusion

Snakebites require rapid and appropriate care. The local population often resorts to traditional treatments, which leads to delays in seeking medical care and the onset of serious complications. Antivenom immunotherapy is the only alternative if administered as soon as possible after the bite. Policies must be implemented to make it accessible in all healthcare facilities and reduce its cost.

## Ethical considerations

Signed informed consent was obtained from the patient’s father for the publication of his medical data.

## Funding

No funding was received for this study.

## Authors’ contributions

Study Design, Data Collection, and Images: LKA, ETB

Manuscript drafting: LKA, TGA

Manuscript review: ETB, LKA, and TGA

All authors have read and approved the final version of the manuscript.

## Conflicts of interest

The authors declare no conflicts of interest.
